# Increasing Positive Perception of Disability Through Depictions of Animals with Disabilities

**DOI:** 10.3390/ani15131861

**Published:** 2025-06-24

**Authors:** Cameron T. Whitley, Marta Burnet, Em Sherwood, Denny Dulaney, Alexander Jones, Courtney Cordova, Emma Hindes, Katya Ankoudinova, Brooklyn Wehr, Corin Yates, Brooke Tucker, Tut Fuentevilla, Caitlin Allessi, Tess Busch, Kevin Kollar, Michelle Hanenburg, Natalie Stier

**Affiliations:** 1 Department of Sociology, Western Washington University, Bellingham, WA 98225, USA; sherwom@wwu.edu (E.S.); emma.e.hines@gmail.com (E.H.); katyaank2003@gmail.com (K.A.); wehrb@wwu.edu (B.W.); yatesc@wwu.edu (C.Y.); 2 Woodland Park Zoo, Seattle, WA 98103, USA; marta.burnet@zoo.org (M.B.); alexander.jones@zoo.org (A.J.); 3 Henry Vilas Zoo, Madison, WI 53715, USA; denny.dulaney@gmail.com (D.D.); cordova.courtney@henryvilaszoo.gov (C.C.); 4 Zoo Montana, Billings, MT 59106, USA; btucker@zoomontana.org; 5 Grizzly & Wolf Discovery Center, Yellowstone, MT 59758, USA; tutf@grizzlydiscoveryctr.com; 6 Como Park Zoo and Conservatory, St. Paul, MN 55103, USA; caitlin.allessi@ci.stpaul.mn.us; 7 Denver Zoo Conservation Alliance, Denver, CO 80205, USA; tbusch@denverzoo.org; 8 Columbus Zoo & Aquarium, Columbus, OH 43065, USA; kevin.kollar@columbuszoo.org; 9 Alaska SeaLife Center, Seward, AK 99664, USA; michelle.hanenburg@gmail.com; 10 Utah’s Hogle Zoo, Salt Lake City, UT 84108, USA; nstier@hoglezoo.org

**Keywords:** animals with disability, zoos, aquariums, people with disabilities, disability in conservation, strategic anthropomorphism, animal well-being, animal welfare, critical anthropormophism, visitor experience

## Abstract

Across the globe, many animals with disabilities live in zoos and aquariums, yet these institutions often face difficult questions about how to share those stories with the public in a way that raises awareness and minimizes concern about the animals’ well-being. This study explored whether anthropomorphic narrative signage could help visitors form meaningful emotional connections with these animals and, in doing so, encourage greater understanding and support for people with disabilities. Drawing on ideas from contact and transfer theories, we conducted observational research in zoo settings, comparing visitor reactions to no signage, detailed signage, and simple signage. We found that simple signage resulted in fewer negative comments, fewer questions about the animal’s health, and less frustration directed at the organization. In a related survey experiment, participants who saw a photo and brief story about an animal with a disability were more likely to express empathy and hold more positive views toward both animals and people with disabilities. These findings highlight the power of accessible messaging in informal learning spaces to reduce stigma and promote inclusion. The project was led by a diverse, interdisciplinary team of zoo professionals and disability scholars, most of whom bring personal experience with physical or cognitive disabilities to the work.

## Simple Summary

Zoos and aquariums around the world care for many animals with disabilities, but staff often face challenges in how to share these stories with visitors. This study looked at whether using simple, narrative signs could help the public connect with these animals and develop more understanding attitudes toward people with disabilities. Researchers observed how visitors reacted to animals when there was no sign, a detailed sign, or a simple one. The simple signs led to fewer negative comments and less concern about the animals’ well-being. In addition, in an online survey experiment, participants who saw a photo and short story about an animal with a disability reported feeling more emotionally connected and more positive toward both animals and people with disabilities. The findings show that clear, thoughtful communication can encourage empathy and reduce stigma. Led by a team of disability scholars and zoo professionals, many with personal experience of disability, this work offers practical guidance for creating more inclusive public spaces.

## 1. Introduction

Thousands of animals with disabilities are living in zoos, aquariums, and sanctuaries worldwide, with several becoming internet sensations. Perhaps one of the most famous is Basil, a Virginia opossum who arrived at the Smithsonian’s National Zoo & Conservation Biology Institute (also known as the National Zoo) in Washington, DC, USA, in 2023. In what may have been a predator attack, Basil sustained several injuries including the loss of an eye. He was picked up in the Washington, D.C. suburbs and taken to a wildlife rehabilitation center. After receiving care, it was determined that he would not be able to survive in the wild and he was transferred to the National Zoo. Basil’s story, filled with survival narratives and human empathy, circulated among numerous news and pop culture outlets [1,2], including The Atlantic [3] and the Today Show, where he was described as a curious, mellow, and friendly creature who enjoys being cozy [4]. Not only did the focus on Basil raise awareness about Virginia opossums, which are often seen as a nuisance or pest [5], but it also increased conversation and coverage about animals with disabilities. The National Zoo introduced Basil to the public not as a suffering animal, but as a thriving, charismatic creature that shares some similarities with humans, and who happens to have a physical disability. Depictions of his disability and the hardships he faces were not lengthy, and the focus was not on misery or suffering. Intentionally or unintentionally this approach seemed to captivate viewers, enhance connections, and promote empathy.

Basil’s story shows the power of animals with disabilities to captivate attention, but did this story change what people thought about animals and people with disabilities broadly? This question is particularly important as research suggests that 70 percent of people feel uncomfortable around people with disabilities and that this discomfort comes from a lack of exposure and ignorance, which leads to the fear of saying the wrong thing or being insensitive when interacting with people with disabilities [6]. In addition, the importance of animals with disabilities and their needs in society is an understudied topic [7]. Contact hypothesis (also known as Intergroup Contact) theory suggests that increased contact with individuals and communities we are unfamiliar with or feel uncomfortable around can reduce negative evaluations and prejudice and increase positive perceptions [8]. It is considered one of the most effective ways to reduce prejudice between groups [9,10]. So, if people lack exposure to people with disabilities, leading to ignorance and discrimination, how can exposure and communication around disability be increased? Similarly, transfer theory suggests that positive engagement in one area may transfer over to our perceptions of other people and events in another area of our lives. So, if people can have positive experiences with animals with disabilities, will the memory and perceptions of these experiences transfer to experiences with other humans and nonhuman animals with disabilities? Both theories are important in describing how perceptions of animals with disabilities may enhance or hinder perceptions of people with disabilities.

One way to increase exposure, encourage discussion, and promote acceptance may be through engaging people with animals with disabilities in informal educational environments like zoos, aquariums, and museums. As demonstrated with Basil’s story, stories of individual animals can have profound impacts on individuals and communities and may have the power to change perceptions. The idea of using animals to help people to better understand difference is not new. Animals have often been used as characters in literature to aid people in navigating and practicing language around sensitive or complex topics [11,12,13]. The research suggests that these depictions are helpful in shaping attitudes at least among children, supporting the contact hypothesis. Specifically, depictions of characters (human and nonhuman) with disabilities in public education influence perceptions of people with disabilities [14]. However, positive depictions of characters with disabilities in literature remain limited [15,16]. Based on the work mentioned above, negative or dramatized depictions of animals with disabilities could have a harmful effect on perceptions not just of the individual animal and disability, but also on animal well-being in general. In some cases, people may conclude that the animal acquired their disabilities in the care of the animal-oriented organization and/or that they would be better off euthanized. In turn, these attitudes could influence the value people place on people with disabilities. The research on these relationships is limited.

In a recent paper, Whitley and colleagues [17] assert that animals with disabilities in informal educational environments could be used as strategic tools to promote greater awareness and support for disability perceptions. The authors present guidelines for talking about animals with disabilities that parallel the Americans with Disabilities National Network Information, Guidance, and Training on the Americans with Disabilities Act [18] guidelines for talking about people with disabilities. Their goal was to encourage people to consider the messages they are intentionally or unintentionally using when talking about the animals with disabilities in their care, and how these depictions might influence what people think about people with disabilities. They call on researchers and practitioners to consider how these guidelines work in practice across different environments with different species and if they can be used to help people better connect to people with disabilities. Heeding this call, we developed a mixed-methods study to assess if adhering to these guidelines produced change in attitudes around animals and people with disabilities.

## 2. Background and Theory

### 2.1. Defining Disability, Ableism, and Speciesism

The consideration of animals with disabilities is important in how we construct our understanding of people with disabilities. Taylor [19] asserts, “animal disability both inspires and horrifies people” as they exist at the intersection of ableism and speciesism (p. 25). Definitions of disability, ableism, and speciesism are numerous. The ADA defines a person with a disability as having a mental or physical impairment that limits one or more major life functions. Although this is one of the most utilized definitions, as it provides statutory obligations, it is inadequate in that it frames disability as an impairment. Adams and colleagues [20] argue, “Disability encompasses a broad range of bodily, cognitive, and sensory differences and capacities. It is more fluid than most other forms of identity in that it can potentially happen to anyone at any time,” (p. 5). This definition situates disability as a difference and not an impairment. However, both definitions lack recognition of nonhuman animals with disabilities. Incorporating nonhuman animals into our understanding of disability is challenging, as much of what we do is to overlay our perceptions of disability onto animal others. As Taylor [19] writes, “we have no idea how other animals comprehend physical or cognitive difference” (p. 24). As Whitley and colleagues [17] assert, despite the challenges, “it is important to define disability among non-human populations both to clarify communication and treatment around these issues and because animals are important symbols in human culture that have the power to alter perceptions and shape behavior” (p. 2). Building on previous work, we use the definition by these scholars, which is attuned to describing disability within human and nonhuman animal populations as a combination of previous definitions and in alignment with the biopsychosocial model of disability [21]:

*Disability refers to an array of cognitive, sensory and physical differences and capabilities that may substantially alter one or more major life functions for human and nonhuman animals either completely or temporarily. It is the result of a complex relationship between an individual’s health conditions and external factors representing the circumstances in which the individual lives.*

Ableism and speciesism are both systems of oppression, with both relying on judging an individual based on perceived limitations, differences, or abilities. According to the Center for Disability Rights [22], ableism is a set of beliefs and practices embedded in culture that normalize the devaluation and discrimination against people with disabilities. Just like disability is socially constructed, so is ableism. In fact, surveys show that Americans do not agree on what does and does not qualify as a disability, especially when it comes to disabilities that are not apparent [23]. In terms of animals, the range of “acceptable” disabilities is even smaller. While visible disabilities are clearly acknowledged, society is also poised to quickly “remove” or euthanize animals with disabilities [24], and our limited knowledge of animal minds has made it so that society does not always recognize psychiatric and cognitive disabilities in animals [25]. This is speciesism in practice. Speciesism is the assumption that humans are superior to animals, and that animals are incapable of the emotions and cognitive processes that humans possess. Researchers are increasingly calling for connections between disability scholars, animal studies scholars, and practitioners to address systematic ableism, speciesism, and the devaluation of disability broadly in conservation [26,27]. Our project addresses this intersection.

### 2.2. Public Perception of People and Animals with Disabilities

Perceptions of people and animals with disabilities are likely connected. Scholars suggest that disability among humans is depicted, erased, and juxtaposed in similar ways as it is among nonhuman animals [28]. People with disabilities are often dehumanized, being rated as having traits and mental capacities similar to nonhuman animals or being less evolved [29]. In a comprehensive review, Wang and colleagues [30] find that the top three factors associated with negative views of people with disabilities were a lack of education about disabilities, lack of contact with people with disabilities, and disability type as being intellectual or intellectual/physical. While there are no formal studies assessing perceptions of animals with disabilities, there are theoretical arguments from scholars suggesting that animals with disabilities are either considered inspirational, tragic, or only worthy of euthanasia [19,31]. These scholars suggest that the value of animals with disabilities is tied to what the animal can provide to humans, so that if there is nothing the animal can provide in terms of inspiration or ability, the animal is no longer deemed useful and should be euthanized [19]. While negative perceptions and misunderstandings of animals with disabilities may lead to the untimely death of an animal, negative perceptions of people with disabilities could lead to social exclusion and isolation [21]. Studies show that with increased education about disabilities [32,33] and greater connections to people with disabilities [34], attitudes can be changed.

### 2.3. Applying Contact Hypothesis and Transfer Theories

Greater exposure to people with disabilities can lead to greater understanding of disabilities and more acceptance [35], and contact and transfer theories can be used to explain this relationship. Contact hypothesis (or contact theory) is a theoretical framework first proposed by Gordon Allport, in 1954, suggesting that positive interactions between members of different groups can reduce prejudice and bias and ultimately improve intergroup connections [8]. It is a social psychological theory, where social engagement leads to changes in how people think about individuals and groups that have different characteristics and identities from themselves. For over 70 years, the limits of this theoretical framework have been tested across disciplines, with initial tests assuming that only direct engagement would produce a significant effect [36,37,38]. As technology has developed and through the recognition of mediated contact as important in developing perspectives, scholars have extended contact hypothesis to include parasocial interaction (PSI), which refers to a one-sided relationship or connection (never an actual direct connection) that develops with another individual, celebrity, or fictional character. PSI has shown promise in changing perspectives on people with differing genders and sexualities [39,40,41], races [42,43], and disabilities [44,45], and those experiencing homelessness [46], among others. Scholars argue that although studies on PSI have expanded, there are a number of unaddressed issues, such as how PSI can be better integrated into society, how PSI varies with different types of figures, and what processes do and do not connect the individual and figure [39,47].

In addition, transfer theory may also be important in shaping attitudes around animals and people with disabilities. Transfer theory suggests that the perceived experiences or emotions in one area of life can influence thoughts and emotions in another area of life [48]. For instance, when individuals are presented with a compelling image and story about an animal with a disability, it may not just impact how they perceive the animal, but it may also carry over into how they perceive people with disabilities. This shift occurs when people recognize emotional and moral parallels between groups, allowing empathy or concern to extend beyond the original subject [49]. In this way, enhancing positive attitudes around animals with disabilities may serve to enhance attitudes for humans with similar disabilities, a process also supported by research where empathy toward one group can increase willingness to support or assist a related group [50].

Few studies apply contact (or PSI) or transfer theories to human–animal relationships. So far studies have focused on how people develop relationships with real and fictional animals, not on how these relationships may alter perceptions of others. An exception is the vast work on how having or engaging with animals at an early age can increase prosocial behaviors [12], although this work does not specifically mention contact theory or PSI and is slightly different because the emotional outcome is not directed at any specific group. Similarly, there is a wealth of literature discussing the importance of visitor and zoo animal interactions in activating emotions and enhancing connections to the environment and others [51,52,53]. For this study, we not only test contact and transfer theories, but we also address some of Bond [39] and Gile’s [47] concerns about the integration of PSI into society (we consider the importance of PSI in zoos and aquariums), how different types of figures work (we use animals with disabilities, an unused group), and what processes work and do not work (we interrogate minimal engagement in an online environment as a baseline starting point).

## 3. Materials and Methods

Data were collected in two phases through exploratory observation and an online survey experiment. We first used an exploratory observation protocol to assess how people were engaging animals with disabilities in exhibits with different types of signage. We then used these findings to inform an online survey experiment to test if images and simple strategic anthropomorphic narratives about real animals with disabilities could influence perception of animals and people with disabilities. All data were collected in 2024. The corresponding author’s institutional review board was consulted on all components of this study.

### 3.1. Exploratory Observation

As a first step, we wanted to understand how people engaged with animals with disabilities in zoo and aquarium environments based on different signage conditions that did or did not explicitly address the animal’s disability. We identified three signage conditions used in zoo and aquarium environments to discuss animals with disabilities: detailed signage, simple signage, and no signage. The detailed and simple signage anthropomorphized the animals, giving the animal a name, pronouns, and discussing their personality. The detailed signage discussed the animal’s disability in length, detailing exactly how the disability was acquired and the past, present, and future impact of the disability on the animal’s overall well-being. The simple signage noted that one animal (using the animal’s name) within a habitat has a disability and that he/she might move or look different, but that this is a normal adaptation for the disability. These statements often noted that the habitat had been altered/adapted to the animal’s needs. The last condition was no signage. The no signage category reflects the fact that some zoos and aquariums opt to not use signage to identify animals with disabilities. We do not provide examples, to maintain the anomality of the organizations that participated.

With the help of zoo and aquarium staff, we identified an animal with a visible disability with each of the signage conditions (no signage, detailed signage, simple signage) across three zoos. Ideally, we would have been able to observe the same type of animal across the three conditions. This was not possible given that we also needed to find a popular animal that could draw a sufficient crowd for observation. For the no signage condition, we observed guests viewing a marine mammal habitat. For the detailed signage condition, we observed guests viewing a bird habitat. For the simple signage condition, we observed guests viewing a land mammal habitat. All three animals could be classified as charismatic. As depicted previously in the story about Basil, animals with disabilities can garner increased attention, and their depiction/perception of treatment can impact perceptions of the organization in terms of animal well-being. Because of this, we will not disclose the exact animal, their name, or the name of the organizations that we partnered with. For each condition, we observed people viewing the habitat and reading the associated signage. We chose weekend observation sessions to optimize the diversity in viewership and limited our observational sessions to the same two hours across each condition/environment (10:00 a.m. to 12:00 p.m.), locating ourselves near the habitats we were observing so we could overhear conversations. Observation of visitors is a widely accepted form of data gathering in zoo and aquarium environments. We following similar processes as described by other scholars [53,54]. We took notes on small 3 × 5 inch notepads instead of clipboards and wore casual clothing so that we could more easily blend into the crowd. We focused on coding the first comment people made about the animal, if they made a comment. As an exploratory observational study, our coding system was not complex. We did three things. We recorded (1) the number of people who viewed the exhibit in two hours, (2) their initial comment or question about the animal’s disability, as being positive, neutral, or negative, and (3) if they mentioned the animal’s disability. We also wrote down as many initial comments as possible to reflect each of the three categories. We did not count infants or children who appeared younger than five. The same two coders were engaged in this process in all organizational settings. In terms of intercoder reliability, we had 100 percent congruence on the number of people visiting the exhibit, 94 percent congruence on people pointing out or mentioning the animal with the disability, 92 percent congruence on negative statements, and 97 percent congruence on positive statements. The exceptionally high intercoder reliability is likely because of the simplistic protocol and the fact that we were stationed relatively close to each other in each setting to overhear the same information. In the results, we present the category counts and example statements. To assess whether the distribution of comments (positive or negative) differed across signage groups, we conducted a Pearson’s Chi-squared test of independence. Group membership (no signage, detailed signage, and simple signage) and comment valence (binary: 0 = negative, 1 = positive) were treated as categorical variables. Following a significant overall Chi-square test, we performed pairwise Chi-square tests comparing each combination of groups. A Bonferroni correction was applied to account for multiple comparisons, adjusting the alpha level to 0.017.

### 3.2. Survey Experiment

In designing the survey experiment, we wanted to make the different animal narrative stories as real as possible. To do this, we partnered with the Advancing Conservation Through Empathy Network (ACE for Wildlife), which facilitates the sharing of knowledge, experiences, and data to drive conservation change through fostering empathy for animals and the environment that sustains them [55]. We crafted an email asking if partner and affiliate zoos and aquariums would be willing to send us images of their animals with disabilities and answer a few questions regarding the animal’s name, gender, disability, interests, dislikes, and personality. Based on previous literature [56], we specifically asked for portraits of the animals. We received images and information on 17 individual animals from ten zoos and aquariums across the Midwest and West.

**Figure 1:**
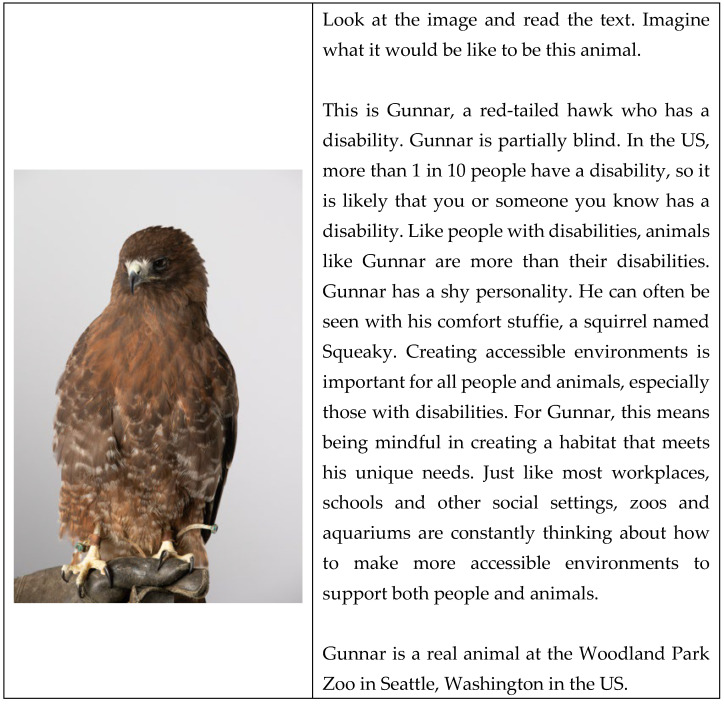
Example of an animal with a disability simple narrative.

The 17 animals could be broken down into four categories: birds (seven individual animals: two screech owls, two bald eagles, a penguin, a red-tailed hawk, a brown pelican), land mammals (six individual animals: an American badger, an African wild dog, a grey wolf, a cougar, a snow leopard, a zebra), sea mammals (three individual animals: two harbor seals, a manatee), and reptiles (one individual animal: a hawkbill turtle). The disabilities the animals had could be broken down into four categories: partial or full blindness (seven animals), amputated limbs (four animals), traumatic brain injuries or hydrocephalus and other apparent disabilities (hunched back, wing injury, limp, angel wing syndrome) (four animals). We used the information provided by the organizations to create short story narratives about each animal. The narratives followed the guidelines for talking about animals with disabilities [17], and the short description format found to be most effective in our exploratory observation. All narratives were similar in length with approximately 140 words and used the same format by introducing the animal, mentioning animal type, acknowledging the disability the animal has, making a comparison to humans, focusing on the animal’s preferences and personality, and ending by mentioning the habitat is accessible and that zoos and aquariums are constantly working on creating environments that promote the well-being of all. For an example of a narrative, see Figure 1.

Measuring attitudes toward people with disabilities is inherently challenging. Most animal-related scales are insufficient, focus on one specific disability, or are too long for practical use [57]. Based on previous research, we wanted to get at two key concepts: value and comfort. For the value concept we assess how much value individuals place on people with disabilities in society. For the comfort concept, we assess how comfortable people feel around those with disabilities. There is no research to suggest that people are often uncomfortable around animals with disabilities. In addition, comfort around people with disabilities and comfort around animals with disabilities is likely substantially different. Because of these reasons, we did not include questions to assess comfort around animals with disabilities. For this study we focus on assessing how images and short narratives of real animals with disabilities impacted the value people place on people with disabilities in society (disability value scale), the value people place on animals with disabilities in society (animal disability value scale), and the level of comfort people feel around people with disabilities (disability comfort scale). Based on previous scales measuring attitudes toward people with disabilities [58,59], we developed the three scales to address these concepts that we term the perceptions of human and animal disability scale (PHADS). Through five small pilot survey experiments, we were able to reduce the narratives down to 140 words and scales down to three items each. This was important because we wanted to produce narrative structures and useful scales that informal learning environments could implement easily. To assess construct validity, we report Cronbach alphas. All scales should ideally be above 0.70; one (disability value scale), is just below this cutoff giving it a borderline acceptable status. In past pilot surveys, this scale performed with a Cronbach alpha between 0.68 and 0.079. Scale items and Cronbach alphas can be found in Table 1. We conducted independent *t*-tests to evaluate whether exposure to an image and narrative depicting an animal with a disability influenced participants’ perceptions of both people and animals with disabilities. We further explain both processes and findings in the results section. IRB approval was obtained from the lead author’s institution.

We created an online survey experiment where 1003 people either received an image and narrative about a real animal with a disability (n = 704) or no image or text (n = 299). All participants were asked about their perceptions of humans and animals with disabilities (PHADS) regardless of them receiving an image or text. There were 17 different animal frames that were presented. This equates to about 41 people seeing each of the 17 animals. The test and control samples are different sizes, as the control survey included items at the end that were used for another study which needed 300 participants. We used Prolific to obtain our sample which is representative based on US Census characteristics. The median time to complete the survey was 4.68 min. People were paid $1 to complete the survey, which equates to about $12 per hour. Once we reached 1000 participants, we stopped the survey to assess results. We used Stata 16.1 and R Studio 12.1 to conduct the statistical analysis.

## 4. Results

### 4.1. Exploratory Observation Results

For the exploratory observation phase of our project, we assessed how people responded to different types of signage about animals with disabilities in zoos. We looked at three types of signage: detailed signage, simple signage, no signage. All three zoos that we conducted observation at are part of the Advancing Conservation through Empathy Network, a larger network of zoos and aquariums interested in enhancing empathy for animals [55]. The simple signage and the detailed signage both anthropomorphized the animals with names, pronouns, and depictions of personality. We used a Chi-square test to interpret the association between signage seen and positive/negative comments. The Chi-square test was significant. Pairwise Chi-square tests revealed significant differences between all three signage group combinations. The no signage group had significantly more negative comments than both detailed signage (X^2^ = 25.19, *p* < 0.001) and simple signage (X^2^ = 86.17, *p* < 0.001) groups. People seeing the simple signage gave significantly more positive comments than those that saw detailed signage (X^2^ = 22.77, *p* < 0.001). All results remained significant after Bonferroni correction (adjusted alpha = 0.017), indicating robust group differences in comment valence. The results can be found in Table 2.

### 4.2. No Signage Observation Results

In a two-hour period, a total of 144 people visited the marine mammal with a disability. There was no signage that mentioned his disability, which impacted his movement. As people watched him move around the exhibit, 118 (82%) commented or posed questions about the disability and/or well-being of the animal. Of these comments and questions, most 102 (86%) were negative. Not only did these comments show concern for the animal, such as “Should we tell someone [the animal] is hurt?” or “I’m worried that he is in pain,” but they were also critical of the animal’s well-being within the organization with statements like, “why isn’t the zoo doing something to help him?” and “I hate zoos because they just leave animals like this.” We coded 16 statements or questions about the animal’s disability as positive or neutral, which included things like, “He is too cute to be neglected,” “I wonder how he eats in that condition?”, and “Do lots of animals in the zoo have a disability?” None of the comments or questions people had about the animal or organization in relation to the visual disability were completely positive or made parallels between human and animal experience.

### 4.3. Detailed Signage Observation Results

In a two-hour observational period, we recorded 94 people visiting the bird with a disability. There was detailed signage about the animal sustaining significant trauma, losing an eye, and having lifelong challenges. The signage was prominent and easy to access. A total of 76 people (81%) made a comment about the animal’s disability after looking at the animal and/or reading the signage. Assessing the welfare of the animal seemed to be a focal point of interest. The comment and questions about the animal’s disability were mixed with 41 comments (54%) leaning negative and 35 (46%) of comments leaning neutral or positive.

Negative comments and questions included concern for the animal, “I just feel bad for him,” “Is he in pain?”, “Is he happy, I just don’t know if I would want to live like that” and “How long has he been like that?” It also included people questioning if the zoo was doing enough, “I guess the zoo is doing everything they can.” Or, “I read the story, but what is the zoo doing for him now?” Some people felt strongly about animal suffering and, although the animal did not appear to be suffering, reflecting on the lengthy narrative, they said things like, “he shouldn’t be kept alive for people to watch him suffer.” Most of the positive comments focused on the animal being an inspiration with things like, “Wow! [animal] has really been through a lot. That’s inspiring” and “He seems to be thriving despite the [disability].” One comment had a neutral tone and made a parallel connection to humans, “Is this like how Uncle [redacted name] got his leg removed?”

### 4.4. Simple Signage Observation Results

In the two-hour observational period, we recorded 106 people visiting the land mammal with a disability. The simplistic sign mentioned that one of the animals in the habitat had a movement-related disability due to an accident, but that he had adapted to the disability. It then listed information about the animal’s personality (preferences), and what the zoo was doing to create an accessible environment. It did not list extensive information about the accident. As with the detailed signage, the signage was centered and easy to approach. A total of 54 people (51%) made a comment about the animal’s disability. Nearly all 47 (87%) of these comments were positive or neutral, often focusing on making connections between the animal’s disability and human experiences. For instance, people said things like, “He has the same thing as my dad,” “[redacted name] (family member) moves like that too,” “It looks like his buddies don’t even know he moves different.” Positive comments about how the organization approaches animal well-being were frequent with things like, “I like how the zoo is taking care of [the animal’s name],” and “This is how those with disabilities should be treated.” There were only seven (13%) negative comments or questions recorded, but most were because someone had not read the sign such as, “What is wrong with the [animal]?” This statement was met with “You dumb a**. Didn’t you see the sign?” Although we did not record general commentary about the animal, there was a clear difference in focus on the animal’s disability and perception of animal well-being in the no signage and detailed signage exhibits. In the simplistic signage habitats, people focused on other aspects of the animal beyond his disability, with statements like, “Look at his eyes, he is so expressive,” and “I love playing like that too”.

### 4.5. Survey Experiment Results

Following the initial exploratory observation phase, we designed an online survey experiment to examine whether exposure to an image of an animal with a disability, accompanied by a brief narrative, would influence participant’s valuation of animals with disabilities, attitudes toward people with disabilities, and their comfort around individuals with disabilities. To measure these outcomes, we employed the PHADS scale that we specifically created for this study. Levene’s test indicated unequal variances between groups; therefore, we conducted two-sample *t*-tests with unequal variance using the Satterthwaite approximation. Results showed significant differences between participants exposed to the image and narrative and those in the control group (who received no image or text), with effects observed consistently across all three disability-related outcome measures. The mean scores for valuing people with disabilities (disability value scale) were nearly a quarter of a point higher (0.23 points) on a seven-point Likert scale for people who received an image and simple text (M = 6.07, SD = 0.04), compared to people who received nothing (M = 5.84, SD = 0.06), −3.05 (508.36), *p* = 0.002. A similar trend emerged for valuing animals with disabilities (animal disability scale) where average value scores were 0.34 points higher on a seven-point Likert scale for those who received an image and text (M = 5.76, SD = 0.44), compared to those who did not (M = 5.42, SD = 0.07), −3.95 (523.13), *p* < 0.001. Seeing an animal with a disability with a simple narrative also had a significant impact on a person’s comfort around people with disabilities. Compared to those who did not receive an image and text (M = 5.38, SD = 0.08), average comfort scores were 0.18 points higher for those who did on a seven-point Likert scale (M = 5.56, SD = 0.05), −2.08 (539.58), *p* < 0.04. Seeing any significant positive change with a quick experimental implementation is promising. If changes in perception of people and animals with disabilities can occur in an online experiment where people engage in content for less than one minute, there are infinite possibilities for real and sustained change to occur through informal educational environments such as zoos and aquariums. Given that we used a variety of animals with a variety of disabilities, we assessed differences across animal groups (birds, land mammals, marine mammals, and reptiles) and across disability categories: partial or full blindness, amputated limbs, traumatic brain injuries or hydrocephalus, and other apparent disabilities. No significance was found in any of these assessments. This suggests that the narratives largely performed the same across animals and disabilities and likely helped people overcome animal bias.

## 5. Conclusions

Informal learning environments like zoos and aquariums are key members in supporting animals with disabilities. However, organizations often struggle with how to talk about the disabilities of their animals in ways that promote awareness and diminish well-being concerns. Part of the task of zoos and aquariums as well as other organizations engaging with animals with disabilities is to resist the devaluation of animals with disabilities, while also trying not to sensationalize and fetishize disability [60]. In this study, we assessed how people engaged different forms of signage related to an animal’s disability. Simple signage that noted the disability, but did not give extensive details, produced the most favorable responses. Building on the exploratory observation, we tested parasocial contact within contact hypothesis to assess if using animal images and simple statements about the animal’s disability with personality features could alter perception of people and animals with disabilities more broadly. We found support for parasocial contact in two ways. The simple statements about an animal with a disability not only initiated more positive comments in person, but in the online experiment it also led to positive increases in perceptions of people and animals with disabilities as having value in society. Viewing the images and simple statements also reduced perception of discomfort around people with disabilities. Finding positive increases in perception of people with disabilities and reduced discomfort around people with disabilities after viewing the animal image and simple anthropomorphic text provides support for the application of transfer theory.

Results from these simple exploratory studies demonstrate that increasing awareness and promoting acceptance of animals and people with disabilities does not need to be complicated or overly time-consuming. Given that the two central issues with people valuing and feeling comfortable around people with disabilities are education and contact, the use of animals could be particularly powerful in connecting people to others with disabilities and practicing appropriate language. Animals have long been used in society, and specifically children’s literature, to address complex topics [61,62]. These texts often evoke emotion and elicit engagement, while also giving space for readers to learn and play with concepts and language that might be new or different. Using animals to discuss disabilities may have a similar impact, giving space to learn about and practice concepts before applying them to people.

This study has some limitations and opportunities for future research. The observational phase of this study was exploratory. We did not observe people engaging with the same animal, species, or within the same organization. A future study could test different signage using the same animal to better control for variation in the environment. The survey experiment only includes birds, mammals (marine and land), and one reptile. While the findings for these groups are interesting, they do not speak to how these narratives might work on animals with less human-like characteristics such as amphibians, fish, and animals without faces. Additional work should be done to assess how these frameworks could be applied to animals with less human-like characteristics. The work in this study largely focuses on highly visible disabilities. Future studies should address public response to less apparent disabilities among animals including intellectual disabilities. Beyond observational work in zoo and aquarium settings, future work could survey people on their perceptions of animals and people with disabilities in other informal learning environments. This work assesses perception at one point in time; additional work could look at assessing perceptions over time to see if positive engagements had a sustained impact. Finally, the PHADS scale performed adequately well. Continued modifications and enhancements should occur to better address different perceptions of disability. This study was designed to engage a deeper conversation about the perceived connections between people and animals with disabilities, to assess if tailored narratives about animals with disabilities could increase perceived value and connection to animals and people with disabilities. Ultimately, this study shows that how we talk about and present animals with disabilities affects not only how people value animals with disabilities, but how they value and feel comfort around people with disabilities. This study opens the door for limitless possibilities for promoting positive attitudinal change around disability, shifting how disability is taught, and potentially changing disability perceptions within and outside of the zoo, aquarium, and animal-focused informal learning environments.

## Figures and Tables

**Table 1:** Perceptions of human and animal disability scale (PHADS) Items.

Scale Items	Cronbach Alpha’s
Disability Value Scale	0.67
People with disabilities are a burden on society (R).	
Society should value people with disabilities more.	
Too many resources are allocated to people with disabilities (R).	
Animal Disability Value Scale	0.71
Animals with disabilities are a burden to society (R).	
Animals with disabilities have a purpose in society.	
All animals with disabilities should be euthanized (R).	
Disability Comfort Scale	0.79
I feel uneasy communicating with people with disabilities (R).	
I feel comfortable around people with disabilities.	
I find it hard to connect to people with disabilities (R).	
R indicates reverse coding.	

**Table 2:** Details of visitors to selected exhibits with animals with disabilities (n = 344).

Signage	Visitors	Comments on Animal’s Disability	Positive or Neutral Comments	Negative Comments
No signage	144	118 (82%)	16 (14%)	102 (86%)
Detailed signage	94	76 (81%)	35 (46%)	41 (54%)
Simple signage	106	54 (51%)	47 (87%)	7 (13%)

## Data Availability

The raw data supporting the conclusions of this article will be made available by the authors on request.
